# Working in fours: generational communication in the emergency department

**DOI:** 10.1186/s12245-023-00536-7

**Published:** 2023-09-13

**Authors:** Mindi Guptill, Ellen Reibling, Tammy Phan, Bryant Khoo, Stephen Lin, Corbin Donham, Cheryl Wang, E. Lea Walters

**Affiliations:** 1 Department of Emergency Medicine, Loma Linda University School of Medicine, Loma Linda, CA USA; 2 Pomona Valley Hospital Medical Center, Pomona, CA USA; 3 Western Michigan University Homer Stryker MD School of Medicine, Kalamazoo, MI USA; 4 Loma Linda University School of Medicine, Loma Linda, CA USA

**Keywords:** Generation, Communication, Emergency department

## Abstract

**Background:**

This study examined the conflicts between different generations working in US emergency departments (ED). We sought to record generational differences involving communication preferences, perceived areas of conflict, work motivations, and attitudes regarding work-life balance.

**Methods:**

We developed a survey to assess the physician perspective on generational conflict in the ED. The survey was distributed to members of the American College of Emergency Physicians, a professional organization comprising emergency medicine physicians in the USA.

**Results:**

We received 696 completed responses. Men represented 60% of respondents and the largest proportion of respondents were emergency physicians working in community settings (53%); 11% were residents. Generation representation was smallest for Traditionalist (2%) and largest for Gen X (43%). Seventy percent reported observing conflict due to generational communication with the largest frequency being once a week (26%). In the associated open-ended questions, 247 (33%) provided 316 anecdotal descriptions of observed conflict. Responses clustered into seven themes (ordered by frequency): Work Ethic, Treatment Approach, Technology Application, Entitlement, Professionalism, Work Life/Balance, and Communication Style. Comparing Work Ethic responses, 52–70-year-olds reported that younger providers are less interested in “accomplishing anything” while 26–34-year-olds resented that attitude. Respondents completing the open-ended questions regarding preventing and responding to conflict provided some insight into helpful strategies including actions supportive of clear communication and standardized policies and expectations. Only 5% of respondents reported that they had discussed generational communication in department meetings with the odds of a woman reporting conflict being less than males (*p* = .01).

**Conclusion:**

Conflicts in the ED in the USA can be attributed to how an individual views the values of someone from another generation. Understanding the frequency and areas of generational conflict in the ED can help medical leaders find strategies to mitigate negative workplace interactions.

**Supplementary Information:**

The online version contains supplementary material available at 10.1186/s12245-023-00536-7.

## Introduction

The present-day emergency medicine (EM) workforce in the USA is composed of four generations of physicians, each different in their perspectives, values, and attitudes. A generation is defined as “a group of individuals born and living contemporaneously” with geography significantly influencing the formation of generational culture, beliefs, and behavior [1]. In the USA, the generations shared contemporary political and social events while experiencing similar parenting styles, which results in comparable personal and professional values, work ethic, communication preferences, and leadership styles [2, 3]. Western or US generational models cannot be applied to the global EM workforce as each country’s unique social, political, and economic events shaped today’s adults. This study examined the conflicts between different generations working in US emergency departments [4]. The US generations included in this study are popularly known as the Silent Generation/Traditionalists, Baby Boomers, Generation X, and the Millennials or Gen Y [5]. Gen Z is the newest generation entering the workforce [6]. Generational differences are known to impact the healthcare environment in the US by creating tension and conflict when values differ [7, 8].

### US generations overview: who are they?

The four generations are generally classified by birth year and defined by the significant historic events that took place during their lifetime [2, 5, 7, 8]. The *Silent Generation* (also known as Traditionalists) includes those born between 1925 and 1945 who were greatly influenced by the Great Depression and post-WWII recovery. They are often described as loyal, hierarchal, patriotic, and altruistic while placing a high value on work ethic, respecting authority, and remaining loyal to an employer [2, 4]. *Baby Boomers* were born between 1945 to 1964 and were shaped by an era of post-war prosperity, the Civil Rights Movement, women’s movements, and the Vietnam War. They tend to be workaholics who sacrifice personal life for professional advancement but differ from the Silent Generation by exhibiting rebellious attitudes toward authority [5, 9]. *Generation X* refers to those born between 1964 and 1980 who were influenced by events like the Cold War, AIDS epidemic, and rising divorce rates. “Gen Xers” were the first of the “latchkey kids” and therefore are often described as independent, questioning authority, and more casual in their approach to work and life [5, 7]. *Millennials* (also known as Generation Y) were born between 1980 and 2000 and experienced the tragedy of 9/11 with ongoing threats of terrorism, the Iraq and Afghanistan wars, and the globalization of business and economics. They were raised in child-centered families while always having technology available to them. They are described as more globally aware, team-oriented, and collaborative. Millennials are also described as having poor work ethic, flexibility fixation, low job loyalty, and difficulties with face-to-face communication [1, 2, 5, 7]. Gen Z is the newest generation, but they did not join the medical workforce until after our data was collected [6].

There are about 938,980 active physicians in the USA, with emergency physicians (EPs) numbering about 45,000. Of EPs, 35% are age 55 or older (Traditionalists and Baby Boomers) and 65% are younger than 55. There are about 8,000 EM residents and fellows [10].

There is a significant amount of research addressing generational diversity and communication in healthcare; however, little research has been conducted categorizing the specific tensions attributed to generational communication conflict in the emergency department (ED). These generational characteristics differ enough to cause misunderstandings that in turn can interfere with patient care goals and department administrative functioning [8]. The importance of reducing workplace conflicts in the ED cannot be understated; teamwork is essential to patient care [11]. Understanding and respecting the generational values can mitigate these conflicts and frustrations and is essential for collaboration and teamwork [7, 12].

The goal of this study is to better characterize generational workplace conflict in the US ED. We believe there are generational differences involving communication preferences, perceived areas of conflict, and attitudes regarding work-life balance that contribute to workplace conflict. To effectively lead in the ED, physicians should understand generational differences to help guide interpersonal interactions across generations [13].

## Methods

### Aim, design, and setting

This was a cross-sectional survey study approved by the Loma Linda University Institutional Review Board and the American College of Emergency Physicians (ACEP) Board. ACEP is a professional organization comprising emergency medicine physicians in the US. This organization was founded in 1968 by a group of 8 physicians dedicated to improving the quality of emergency care. The project was supported by a section grant awarded by ACEP to the American Association of Women Emergency Physicians (AAWEP).

### Study setting and population

We distributed a survey to 35,658 ACEP members. The survey instrument was drafted by the authors, shared with a convenience sample of AAWEP members, and modified per their feedback. It was then piloted to a separate sample of 8 EPs and refined again. The final survey instrument was produced in electronic (Qualtrics Inc., Provo, UT) format. The survey is attached as a supplementary file.

### Study protocol

The ACEP central office distributed the electronic survey link on behalf of the authors in late 2016, followed by three reminders over a 10-week period. The survey took an average of sixteen minutes to complete and was anonymous.

### Key outcome measures

Our primary outcome measure was the frequency of observations attributed to generational conflict as defined by respondents. Secondary outcomes were the type of conflict, observations of how the conflict was resolved, suggestions for preventing identified issues, and frequency this issue was discussed in department meetings.

### Data analysis

We descriptively report all results using STATA 15.1 (StataCorp, College Station, TX). Open-ended questions regarding conflict anecdotes were coded by two independent coders, who are both authors on this manuscript. Response coding was refined through an iterative design process comparing coding agreement to achieve consistent category assignment [14]. Codes were routinely reevaluated to ensure consistency and to identify codes needing clarification. After all comments were analyzed, the authors met four times to summarize codes into major themes and identify exemplary quotes relevant to each theme, and any discrepancies were resolved by discussion.

## Results

### Subject characteristics

We received 696 completed responses. The response rate for this study was 2%. Men represented 60% of respondents and the largest proportion of respondents were EPs working in community settings (53%); 11% were residents. Generation representation was smallest for Traditionalist (2%) and largest for Gen X (43%). Our survey did not include race or nationality demographic data but focused on the age and sex of respondents See Table 1.

**Table 1:** Respondent characteristics (*n*/%)

	Generations				
	Millennial	Gen X	Baby Boomer	Traditionalist	Total
Gender^1^					
Male	73 (11)	154 (23)	159 (24)	13 (2)	399 (60)
Female	68 (10)	133 (20)	62 (9)	*	264 (40)
Total	141 (21)	287 (43)	221 (33)	14 (2)	663
Role^2^					
Academic EP	38 (6)	94 (14)	52 (8)	*	184 (26)
Community EP	37 (5)	179 (26)	150 (22)	6 (1)	372 (53)
Residents	65 (9)	14 (2)	*	*	79 (11)
Other	7 (1)	11 (2)	23 (4)	*	44 (6)
Total	147 (21)	299 (43)	235 (34)	15 (2)	696

Percentages may not add up to 100 because of rounding, *notes cell frequency < 5

^1^Pearson chi^2^(5) = 28.26, *p* < .001; ^2^Pearson chi^2^ (15) = 332.30, *p* < .001

### Main results

When asked about conflict, 70% reported observing conflict due to generational communication. The largest frequency was once a week (26%), followed by daily (20%) and 2–3 × /week (17%). Responses by gender differed significantly overall, but not by generation or role. Table 2 details these results.

**Table 2:** Conflict observation (*n*/%)

	Observed conflict due to generational communication		
	Yes	No	Total
Generation^1^			
Millennial	102 (71)	45 (29)	147
Gen X	212 (71)	87 (29)	299
Baby Boomer	160 (68)	74 (32)	234
Traditionalist	9 (56)	7 (44)	16
Total	483 (70)	213 (31)	696
Gender^2^			
Male	259 (65)	138 (45)	397
Female	201 (76)	63 (24)	264
Total	460 (70)	201 (30)	661,661,661
Role^3^			
Academic EP	136 (74)	47 (26)	183
Community EP	254 (68)	118 (32)	372
Residents	51 (41)	28 (59)	125
Other	40 (67)	20 (33)	60
Total	481 (70)	213 (31)	6946

Percentages may not add up to 100 because of rounding

^1^Pearson chi^2^ (3) = 1.74, *p* = .63

^2^Pearson chi^2^ (1) = 8.90,* p* < .01

^3^Pearson chi^2^ (3) = 5.87, *p* = .32

In the associated open-ended questions, 247 (33%) provided 316 anecdotal descriptions of observed conflict. Responses clustered into seven themes (ordered by frequency): Work Ethic (20%), Treatment Approach (19%), Technology Application (16%), Entitlement (12%), Professionalism (12%), Work-Life Balance (11%), and Communication Style (9%). For example, comparing Work Ethic responses, Baby Boomers reported that younger providers are less interested in “accomplishing anything” while Millennials resented that attitude. Table 3 defines each theme, supported by exemplary quotes contrasting responses by generation group.

**Table 3:** Conflict themes definition and illustrations

Category definition	Exemplary quotes	
Work Ethic	Millennial:	Baby Boomer:
• Valuing self over patient	I resent the attitude that residents today are lazy. Older physicians have burned out and don’t do the right thing and take care of patients …Older people cherry picking charts, working slowly	There is a striking difference in apparent work ethic, with many younger workers (nurses more than docs, but both) seeming to be much less interested in actually accomplishing anything and more interested in punching the time clock as compared to older folks. Does my bias show? It’s “not my job” vs “I’ll make sure it gets taken care of…” Younger emergency physicians have bought into the philosophy that lifestyle (i.e., privilege) trumps taking care of patients (i.e., responsibility) Most younger physicians, nurses, ancillary staff do not realize the importance of their job. They take no initiative. They do only what the computer or EHR requires and that’s it
Treatment Approach	Gen X:	Millennial:
• Authoritarian v. patient-centered • Sticking to tradition with lack of evidence	People in the generation right behind mine tend to question everything because they want to understand the right plan for the patient, but people in my generation and especially the generation above me tend to want their orders for a patient followed without question. I have seen this on numerous occasions where an older person asks a younger person to do something for a patient and gets upset when they get a million questions Work ups. New MDs rely heavily on radiological studies. Not physical exams Young practitioners practicing very defensively, high utility of labs, imaging; MDs and nurses driven by protocols and benchmarks (some that they don’t understand)	Older attendings not being receptive to current literature and doing things based on anecdotal evidence. When opinions vary in subjective scenarios and the older generation demands that one way be done because “that’s how it is always done” even with recent evidence to suggest otherwise I have difficulty getting more “old school” consultants on board with a collaborative approach to patient care—they tend to be more hands off
Technology Application	Baby Boomer:	Millennial:
• Underuse or overdependence on technology for patient care and communication • Perception that technology use is replacing patient-centered care • Less patient interaction and dependence on social media to communicate	I am less computer savvy than my younger partners, and thus have more difficulty learning ultrasound, using simulators, learning from “podcasts,” etc I ask another provider “do you remember the dose of ….?” and they pick up a smart phone and google it Everything is done electronically so the newer generation lacks social skill which is so vital to good patient care	Traditionalists can’t give up printing lab values out when they are on the EMR. A senior staff member occasionally submits hand-written requests for our online scheduling program Older ED physicians needing help navigating the EMR/system: putting in the correct orders, diagnoses, etc
Entitlement	GenX:	Traditionalist:
• Expecting individual accommodations • Perception that the younger generations want rewards before they have “put in their time”	Entitlement among residents and/or junior faculty members Residents/junior faculty members craving direct feedback for improvement while older faculty feel uncomfortable providing such feedback	Younger physicians seem to want instant rewards rather than working their way up through a system Trainees wanting the program to adapt to them, rather than their adapting to a new role A medical student wanted time off that would not work with the schedule. She did not seem to understand that she was not special and schedule could not be bent to fit her specific requests I see that some newer people do not hesitate to switch jobs or leave a situation that doesn’t meet every single one of their expectations Lack of understanding that sometimes you have to just “suck it up”
Professionalism	Millennial:	Baby Boomer:
• Patient perception • Problems with credibility • Perceptions of respect	There are lots of little things that come up during the day—one of the most common is an older patient or a patient’s older family member making some remark about the resident or even the attending being “too young to be a doctor” or “too pretty.” The lack of professionalism in terms of timeliness, respect for authority Mostly related to accepted dress in ED	Medical students are not as respectful as we used to have to be—referring to attendings by first name. Older male colleagues will call female residents “blondie.” Younger physicians being poor at communicating with older patients as well as the reverse The older team member did not feel that the younger team member was professional in interacting with patients. The younger team member did not share the same social values held by the older team member. The younger was much more casual when interacting with older patients. The older team member felt this was too casual, calling patients by first names, not addressing them with Ma’am or Sir
Work-Life Balance	Millennial:	Traditionalist:
• Conflict related to staffing the ED • Burnout because of desire to balance work/family life	Told by my female program director that taking more than 6 weeks maternity leave was selfish and unprofessional. She followed by saying that the Hippocratic oath never mentioned family, and that I need to get used to that fact that I’m a doctor now, so my career comes before everything, including my family There are really unspoken conflicts around scheduling, mainly night shifts Younger physicians, often parents, place high value on balance and family time. This is often misinterpreted as laziness or lack of investment in their profession, with negative consequences	Younger emergency physicians have chosen this specialty not for the work but for the time off to indulge their pleasure. They have forgotten that recreation, by definition, is to re-create one to return to their essential task-work Intermediate plans include getting off of night shifts by their 40’s, retire by their 50’s Some of the newer people seem to want much more vacation and more time off than what seems normal. This isn’t a part-time job or a per diem position Lots of conflict on the schedule, working hours, late stay, etc.-often different ideals between older docs (55 +) and younger Docs (mid 30 s) To some of the new grads lifestyle is much more important than professional achievement or income.—in my opinion
Communication Style	Baby Boomer:	Millennial:
• Critical feedback perception • Use of language perceived to be offensive	Whereas traditionally important issues have been discussed in person during a meeting or at least one on one in a phone call more and more young doctors feel comfortable discussing important things via text or via e-mail Direct communication is perceived as anger. Young RN gets told to do a job. She/He perceives the tone of voice to be very aggressive and files a bullying charge Young residents do not take negative feedback well. They look only for positive reinforcement Residents communicate to attendings with text messaging in an informal tone; attendings interpret both the mode of communication and the language used as unprofessional. For example “hey” as a salutation, using all lower caps, using text messaging to let the attending know that they are running late to a shift, using text messaging to inform attendings that they are running late rather than ask for forgiveness for running late Younger generations are more cavalier about electronic communication—text messages in the middle of the night or to communicate operational or leadership issues, multiple phone calls without leaving a VM, preference for IM/text (immediacy) over email. Poor interpersonal communication skills Differences in communication style, particularly with written communication. The specific example is a resident communicating with attendings from other services via email and using improper punctuation, capitalization, grammar, and even emojis. She doesn't understand that work communication and texting with friends require different styles of communication	Younger people prefer to communicate about work via email or text while other generations prefer the phone Different modalities of communication and information gathering—social media vs. email I think this is more related to gender than age, and older men having difficulty with younger female physicians, and speak in a derogatory tone or not agree with management, when it is clearly appropriate, and also not do not take feedback well from a younger physician, especially younger female to older male

### Suggested solutions

Respondents completing the open-ended questions regarding preventing and responding to conflict provided some insight into helpful strategies. These responses are listed in Table 4. Most responses reflected actions supportive of clear communication and standardized policies and expectations. Some comments expressed dismay at not knowing what to do or lamented that the ED environment “forces potential for misunderstandings” that are difficult to resolve thus reinforcing the need for this study.

**Table 4:** Suggested strategies for response and/or prevention

Policy • Document expectations on areas that might generate conflict (e.g., appropriate times to transfer care of critically ill patients, exception to duty hours, phones in work areas) • Take time to remove the conflict from the clinical area and speak to people involved privately • We had group agreement on different work schedules…though not everyone was happy with decision
Training • Develop and/or participate in training on supervising employees including inter-generational considerations such as values, priorities, perceptions of each generation. I think a shorter program could be helpful for emergency department team members—regarding each other and our patients • Practice two-way feedback sessions in conference setting to develop expectations for tone and content
Practice: • Have millennials help write job descriptions to incorporate their perspective • Discuss and listen with respect while trying to understand feedback • Develop a culture of speaking up about generalities to make directions very clear • Overall tone of what is expected and respect for all is instilled from the leadership so all physicians, nurses, management, and ancillary staff including techs and secretaries should be involved in displaying the correct behavior/attitude so that it is ingrained in all

Only 5% of respondents reported that they had discussed generational communication in department meetings.

## Discussion

Our study revealed that most respondents reported generational conflicts regardless of setting type (US academic or community ED). Qualitative analysis of the coded responses revealed clear differences in generational perspectives on conflict and women observed more conflict than men. Each generational cohort brings unique skills, perspectives, and demands that must be acknowledged to maximize communication and productivity in the ED [15]. ED physician leaders need to maintain and strengthen team structures while promoting excellent teamwork and collaborative practice [16].

### Potential conflict areas

Understanding the areas of potential conflict is critical for ED functioning [17]. We identified seven themes: Work Ethic, Treatment Approach, Technology Application, Entitlement, Professionalism, Work Life-Balance, and Communication Style. This is consistent with literature explaining that generational values differ by their attitude towards work. Baby Boomers are often described as “living to work”, while Gen Xers “work to live,” and Millennials “work while living.” [5]. Generation X and Millennials may be perceived as less committed to medicine because, unlike the two previous generations, they tend to favor a work-life balance as opposed to more professional responsibilities [18]. Our respondents agree; when comparing Work Ethic responses, Baby Boomers reported that younger physicians are less interested in “accomplishing anything,” while Millennials resented that attitude. Treatment Approach conflicts centered around “older specialists tend to hold on to outdated beliefs” and perceived resistance to learning new practices. Of our seven conflict themes, conflict in Work-Life balance was unexpectedly rare given previous research done on areas of generational conflict in medicine [9].

### Considerations for emergency department directors and leaders

The role of the ED director is to recognize and solve problems, while considering how hierarchical positions and gender roles may intertwine with conflicts displayed as generational ones. The US ED leadership positions are mostly held by Baby Boomers, while Gen Xers are relegated to midlevel positions [2]. Understanding generational preferences and frames of reference is essential for collaboration and teamwork in the ED [12]. In the US ED setting, reporting of generational conflict is not common. Our study showed that while most people attribute conflict to generational differences, the subject is not discussed in department meetings. Directors should also recognize the potential for generational differences to vary by gender. Our data reveals that women EPs were more likely to report generational conflict than men. Recognition of the common presence of generational conflict in the ED can lead to department conflict mitigation strategies focusing on clear communication with standardized policies and procedures.

Better communication and understanding needs to happen for the ED to function, rather than allowing negative statements or generational stereotypes to fester [19]. Incentive and promotion structure could be optimized with explicit workplace expectations, avoiding vague principles or assumptions [15].

### Strategies to mitigate generational issues in the ED

Generational differences and communication research have been performed among nurses [12, 20] and medical specialties including surgery [21], pediatrics [17], Ob-Gyn [19], anesthesia [8], and neurologists [22]; however, we do not know of another paper describing generational communication conflict in the US ED setting. It is critical that the department director create a work environment that embraces generational differences to maximize effectiveness [9]. Respondents recognized the pitfalls of generational communication in the ED because “clinical urgency/emergency forces potential for misunderstandings.” Recommendations for improved policy, training, and communication practices are outlined in Table 4.

Concrete solutions for generational conflict in the ED are an area for further research. A toolkit “Working with Generations in the Emergency Department” was developed from this research to stimulate discussion about the assets and challenges of working with multiple generations and is freely available online [23]. Curriculum is needed to assist these efforts [24].

### Limitations and further investigation

Our survey is self-reported; however, there may be bias related to people venting negative experiences which underplays the value of the generational workforce. The use of cross-sectional surveys fails to capture the influence of the aging process. Our study may have not adequately accounted for differences due to cultural norms, such as avoiding eye contact to communicate respect, and these issues should be explored in any team effort to address generational conflict. The survey response rate was low (2%), potentially making the sample less representative with sampling bias. Generation representation in our survey was smallest for Traditionalist, thus their voice may not be fully understood. We did not include demographic data related to race and nationality but focused on age and sex of respondents. Further investigation of what ED workplace characteristics are important to each generation, including race and nationality, could provide useful information for administrators tasked with recruitment, retention, and scheduling.

## Conclusion

Generational values and characteristics differ enough to cause misunderstandings that in turn can interfere with patient care goals and administrative functioning of a department. Understanding and respecting the values of four generations working side by side in the ED can mitigate conflicts and frustrations and is essential for collaboration and teamwork. It is critical that department directors create a work environment that embraces generational differences to maximize effectiveness. Concrete solutions for generational conflict in the ED are an area for further research.

### Supplementary Information

**Additional file 1.** 

## Data Availability

The datasets used and/or analyzed during the current study are available from the corresponding author on reasonable request.
