# Supplementary material for: Working in fours: generational communication in the emergency department
Source: Int J Emerg Med. 2023 Sep 13;16:58. doi: 10.1186/s12245-023-00536-7 (PMC10498580; doi:10.1186/s12245-023-00536-7)
Supplement: Supplementary file 1 — Additional file 1. [file 12245_2023_536_MOESM1_ESM.pdf]

Default Question Block

Working in Fours

April, 2016

Dear ACEP Colleague:

We have a hunch and need your input. Our hunch is that intergenerational communication contributes to conflict in clinical workgroups. Your input will help us define the problem and develop a toolkit to address this issue. ACEP has awarded a section grant to AAWEP to support this study.

We estimate it will take about 15-20 minutes to complete the survey. Your participation is voluntary and anonymous.

There is a minimal risk of breach of confidentiality; however, this risk will be minimized by using software that allows you to complete and submit the survey anonymously. The link below will take you to the survey. You may stop answering questions at any time or choose not to submit your answers. When we receive the results, no information will link your answers back to you.

Although you will not benefit directly from this study, the information provided will help us develop a toolkit that will be available via ACEP's website. You may contact an impartial third party not associated with this study regarding any question or complaint by calling 909-558-4647 or e-mailing patientrelations@llu.edu for information and assistance.

Thank you in advance for considering this invitation. If you have any questions, please email me at ewalters@llu.edu.

If you wish to proceed and participate in the survey, please click on the NEXT button provided below. By clicking forward, you are giving your consent to participate.

Sincerely,

Lea Walters, MD
Principal Investigator
Loma Linda University
Department of Emergency Medicine

What is your gender?

- ◯ Male
- ◯ Female

What is your age group?

- ◯ 18 - 25
- ◯ 26 - 34
- ◯ 35 - 44
- ◯ 45 - 51
- ◯ 52 - 70
- ◯ Older than 70

What is your CURRENT and PRIMARY clinical occupation?

- ◯ Academic EP
- ◯ Community EP
- ◯ EM Resident
- ◯ Medical Student
- ◯ Retired EP
- ◯ Other Specialty Physician
- ◯ Other: ______

What other responsibilities do you have? Check all that apply.

- ☐ Research
- ☐ Resident Program management (formal role as Director, Associate Director, etc.)
- ☐ Clerkship management (formal role as Director, etc.)
- ☐ Clinical Program management (formal role as Medical Director, etc.
- ☐ Other: ______

Have you observed conflicts that appeared to be related to differences in generational values or communication styles?

- ◯ Yes
- ◯ No

How often does it occur?

- ◯ Never
- ◯ Less than Once a Month
- ◯ Once a Month
- ◯ 2-3 Times a Month
- ◯ Once a Week
- ◯ 2-3 Times a Week

◯ Daily

Briefly describe one or more examples of what you observed.

Who was involved?

	Role of person involved?	Estimated age of this person?	Gender? Choose one.	
	Click if YES	Type in number	Male	Female
ED Physician	◯		◯	◯
ED Resident	◯		◯	◯
Other Physician	◯		◯	◯
Other Resident	◯		◯	◯
Nurse	◯		◯	◯
Tech	◯		◯	◯
Medical Student	◯		◯	◯
Administrator	◯		◯	◯
Other	◯		◯	◯

Did you observe the resolution to the conflict?

Yes	No
◯	◯

If yes, what resolution did you observe?

To what extent did these people CONTRIBUTE to the conflict?
0 = No contribution to 5=Very strong and essential contribution.

	No contribution	A slight contribution	Definite contribution	Significant contribution	Very strong, essential contribution
ED Physician	○	○	○	○	○
ED Resident	○	○	○	○	○
Other physician	○	○	○	○	○
Other Resident	○	○	○	○	○
Nurse	○	○	○	○	○
Tech	○	○	○	○	○
Medical Student	○	○	○	○	○
Administrator	○	○	○	○	○
Other	○	○	○	○	○

Why do you think the conflict occurred?

To what extent did these people HELP RESOLVE the conflict?
0 = No contribution to 5=Very strong and essential contribution.

	No contribution	A slight contribution	Definite participant in resolution	Significant contribution	Very strong, essential contribution
Ed Physician	○	○	○	○	○
ED Resident	○	○	○	○	○
Other physician	○	○	○	○	○
Other Resident	○	○	○	○	○
Nurse	○	○	○	○	○
Tech	○	○	○	○	○
Medical Student	○	○	○	○	○
Administrator	○	○	○	○	○
Other	○	○	○	○	○

What strategies did people use to resolve the conflict?

What strategy or effort would you suggest to prevent this conflict from occurring again?

How much do you agree with the following statements?

	Strongly Disagree	Disagree	Neither Agree nor Disagree	Agree	Strongly Agree
Keeping up with advances in medical technology is essential to making correct medical decisions.	○	○	○	○	○
Younger people tend to be in a hurry and take too many shortcuts.	○	○	○	○	○
Maturity comes with age and experience.	○	○	○	○	○
I would be comfortable reporting to someone who is younger than myself.	○	○	○	○	○
Years of service are the most important criteria for promotion.	○	○	○	○	○
I learn best by watching others.	○	○	○	○	○
Life is more meaningful with leisure time.	○	○	○	○	○
Nothing is impossible if you work hard enough.	○	○	○	○	○
It is important to stay busy at work and not waste time.	○	○	○	○	○
I consider the opinions of people with more experience as more influential than those newer to the field.	○	○	○	○	○
	**Strongly Disagree**	**Disagree**	**Neither Agree nor Disagree**	**Agree**	**Strongly Agree**
Opportunity for social interaction is a priority at work.	○	○	○	○	○
I feel uneasy when there is little work for me to do.	○	○	○	○	○
I am willing to spend time outside of work to participate in team-building activities with co-workers.	○	○	○	○	○
Outcomes take priority over methods in my work.	○	○	○	○	○
Authority figures always command my respect.	○	○	○	○	○
One should always take personal responsibility for one's actions.	○	○	○	○	○
I ask for help when I do not understand how to use new technology.	○	○	○	○	○
A mentor is essential to succeeding in the workplace.	○	○	○	○	○

	Strongly Disagree	Disagree	Neither Agree nor Disagree	Agree	Strongly Agree
When under stress, it is OK to raise your voice to make a point in the workplace.					
Loyalty to a specific employer is key to success.					

	Strongly Disagree	Disagree	Neither Agree nor Disagree	Agree	Strongly Agree
I devote time outside of work to helping others.					
It is essential to stay up to date with trends in popular culture.					
I work best with leaders who communicate a charismatic mission to nurture pride in the organization.					
Leaders should intervene only when problems arise.					
I appreciate being told what to do with as few words as possible.					
Leaders are most successful when they seek to understand the concerns of individuals.					
I am inspired by leaders who challenge assumptions to look at problems in new ways.					
Leaders call attention to what others can get for what they accomplish.					
Working as a team is the best way to get something done.					
Leaders should ask no more of others than what is absolutely essential.					

	Strongly Disagree	Disagree	Neither Agree nor Disagree	Agree	Strongly Agree
Conflicts are best managed by talking it out with other people.					
The best way to solve a conflict is to focus on a solution.					
I prefer to depend on the strength of relationships I've developed to resolve conflict.					
I prefer to quickly start working on the answer to a problem.					
I appreciate receiving critical feedback to improve my performance.					

On a scale of 1 to 5, how much do the following help motivate you at work?

	Not a motivator	A slight motivator	Somewhat motivating	Strong motivator	Essential motivator
Money/Salary					
Helping others					
New opportunities					
Praise/recognition					
Schedule					
Control/responsibility/authority					
Other					

On a scale of 1 to 5, how much do the following add stress to your work environment?

	No stress	Slight stress	Some stress	Strong stress	Really Significant stress
Lack of support					
Conflict with peers					
Conflict with nurses					
Conflict with other professionals					
Uncertain about treatment decisions					
Workload					
Inadequate preparation					
Death and dying					
Lack of resources					
Not enough time					
Other					

What communication styles do you most value in the workplace?
0=not preferred to 5=most preferred

	Not preferred	Slightly preferred	Preferred	Strongly preferred	Most preferred
Formal					
Polite					
Direct or blunt					
Diplomatic					
Other					

When important information needs to be relayed, I prefer:

Email	Text message	Phone conversation	Face-to-face communication	Written memo
○	○	○	○	○

When general information needs to be relayed, I prefer:

Email	Text message	Phone conversation	Face-to-face communication	Written memo
○	○	○	○	○

Regarding life balance:

I have a balanced life	Demands of work interfere with home and family	Family responsibilities interfere with work	I have enough time to do the things that I want to do
○	○	○	○

I hope to retire at [insert age].

Does your department discuss intergenerational communication?

Yes	No
○	○

Are these discussions formal or informal?

Formal	Informal
○	○

What ideas/programs/resources have you or your department implemented to address generation issues?

Is there anything else you want to tell us about intergenerational communication that we have not asked you already?

Block 1

THANK YOU for completing this survey.
Please encourage your colleagues to complete the survey.
Click on NEXT to submit your answers.
